# Abundance Imparts Evolutionary Constraints of Similar Magnitude on the Buried, Surface, and Disordered Regions of Proteins

**DOI:** 10.3389/fmolb.2021.626729

**Published:** 2021-04-30

**Authors:** Benjamin Dubreuil, Emmanuel D. Levy

**Affiliations:** Department of Structural Biology, Weizmann Institute of Science, Rehovot, Israel

**Keywords:** protein abundance, protein evolution, protein structure, misfolding, intrinsic disorder, contact number, misinteraction, yeast proteome

## Abstract

An understanding of the forces shaping protein conservation is key, both for the fundamental knowledge it represents and to allow for optimal use of evolutionary information in practical applications. Sequence conservation is typically examined at one of two levels. The first is a residue-level, where intra-protein differences are analyzed and the second is a protein-level, where inter-protein differences are studied. At a residue level, we know that solvent-accessibility is a prime determinant of conservation. By inverting this logic, we inferred that disordered regions are slightly more solvent-accessible on average than the most exposed surface residues in domains. By integrating abundance information with evolutionary data within and across proteins, we confirmed a previously reported strong surface-core association in the evolution of structured regions, but we found a comparatively weak association between disordered and structured regions. The facts that disordered and structured regions experience different structural constraints and evolve independently provide a unique setup to examine an outstanding question: why is a protein’s abundance the main determinant of its sequence conservation? Indeed, any structural or biophysical property linked to the abundance-conservation relationship should increase the relative conservation of regions concerned with that property (e.g., disordered residues with mis-interactions, domain residues with misfolding). Surprisingly, however, we found the conservation of disordered and structured regions to increase in equal proportion with abundance. This observation implies that either abundance-related constraints are structure-independent, or multiple constraints apply to different regions and perfectly balance each other.

## Introduction

During the course of evolution, mutations arise throughout genomes and can impact every protein at every site. However, contemplating a multiple sequence alignment of orthologous sequences typically shows widely differing levels of conservation across sites. Additionally, comparing multiple sequence alignments of different orthogroups shows even larger differences: certain groups such as those of ribosomal genes can be well conserved despite hundreds of millions of years of divergence, while others accumulate mutations much faster.

Amino-acid residues within proteins are subject to functional, biophysical, and structural constraints that are interconnected. These constraints result in different degrees of purifying selection along the sequence (i.e., purging of deleterious mutations by natural selection), which yields different levels of positional conservation. We discuss here structural aspects related to these constraints while placing an emphasis on works of Cyrus Chothia, to whom this issue is dedicated, and refer the reader to several reviews for a comprehensive overview (Liberles et al., 2012; Sikosek and Chan, 2014; Echave et al., 2016; Echave and Wilke, 2017). Following the characterization of the first few structures of proteins, their comparative analysis made it clear that the burial of non-polar residues accompanied with Van der Waals interactions and hydrogen bonding were the main contributors to the folding free energy (Chothia, 1974, 1975, 1976; Miller et al., 1987). Confirming the “hydrophobic bonding” intuition of Kauzmann (Kauzmann, 1959) and relying on calculations of molecular surfaces based on the algorithm of Lee and Richards (1971), Chothia estimated that each square Ångstrom of accessible surface removed from contact with water provides a free energy gain of 25 cal. Mol^–1^ (Chothia, 1974, 1975). At the same time, he provided universal relationships governing protein folding, e.g., on the proportion of the total accessible surface of a polypeptide chain that becomes buried upon folding (Chothia, 1975). This simple relationship has a profound meaning with respect to surface-to-volume ratios in folded proteins, notably that longer proteins should fold following a beads-on-a-string model rather than by forming larger beads (Wetlaufer, 1973) – indeed it was soon realized that beads (domains) are fundamental units of protein evolution (Chothia, 1992; Murzin et al., 1995; Bateman et al., 2002; Gough and Chothia, 2002). On top of hydrophobic bonding energy, a high degree of steric complementarity creates a well-packed protein interior (Chothia, 1975), in which mutations are incrementally accommodated by small structural changes (Lesk and Chothia, 1980). Ultimately, as sequences diverge, structures do too, albeit more slowly (Chothia and Lesk, 1986, 1987). Considering that structures are globally maintained during the course of evolution, it is intuitive that buried residues, which contribute to folding and stability more than surface residues (Creighton and Chothia, 1989; Lim and Sauer, 1989; Tokuriki et al., 2007), are more conserved (Koshi and Goldstein, 1995; Goldman et al., 1998; Guo et al., 2004; Bloom et al., 2006; Sasidharan and Chothia, 2007; Goldstein, 2008; Conant and Stadler, 2009; Franzosa and Xia, 2009; Liberles et al., 2012; Yeh et al., 2014; Echave et al., 2015; Shahmoradi and Wilke, 2016; Spielman and Wilke, 2016; Echave and Wilke, 2017; Liu et al., 2017).

We saw that the structure of a protein could help explain why certain positions – notably those buried and in contact with a large number of neighboring residues, are more conserved than others. Protein structure can also help to rationalize why certain proteins, e.g., those with more designable folds, evolve faster than others (Shakhnovich et al., 2005; Bloom et al., 2006). Globally, however, structural information only explains a small fraction of the heterogeneity in evolutionary rates seen across different proteins. Several studies have singled out other protein-centric properties associated with this heterogeneity (Zhang and Yang, 2015), including function (Wall et al., 2005; Lopez-Bigas et al., 2008; Xia et al., 2009), essentiality (Hurst and Smith, 1999; Hirsh and Fraser, 2001; Jordan et al., 2002; Liao et al., 2006), the number of interaction partners (Fraser et al., 2002; Bloom and Adami, 2004; Fraser and Hirsh, 2004; Hahn and Kern, 2005; Kim et al., 2006; Xia et al., 2009), or cellular abundance (Pal et al., 2001; Krylov et al., 2003; Rocha and Danchin, 2004; Subramanian and Kumar, 2004; Drummond et al., 2005; Bloom et al., 2006; Liao et al., 2006; Popescu et al., 2006; Pál et al., 2006; Sällström et al., 2006; Drummond and Wilke, 2008; Xia et al., 2009; Zhang and Yang, 2015). The latter is, by far, the most significant, in particular among unicellular organisms where there is no complexity added by tissue-specific expression. Several mechanistic interpretations of this abundance-conservation association have been proposed (Drummond et al., 2005; Drummond and Wilke, 2008; Cherry, 2010; Gout et al., 2010; Plata et al., 2010; Levy et al., 2012; Yang et al., 2012; Park et al., 2013; Zhang and Yang, 2015) and remain a matter of active debate (Plata and Vitkup, 2018; Razban, 2019). We will scrutinize this relationship further in the results and discussion section, in the context of the results presented.

We have seen how protein structure helped to interpret and rationalize data on evolutionary conservation. Here, we invert this logic to characterize structural properties of disordered regions from data on their evolutionary conservation. First, we compared the evolutionary rate of disordered regions to that of surface residues in the same protein and found that disordered regions are equivalent to super-accessible surface residues. Second, we know that the divergence of surface and core residues is interdependent. In other words, a protein’s surface can hardly diverge without mutations arising in its interior as well, and vice-versa. We confirmed this finding in showing that evolutionary rates of surface and interior regions are correlated within proteins (*R* > 0.85). In contrast, the evolutionary rates of disordered and domain regions were poorly coupled (*R* ∼ 0.25), indicating that disordered regions are, for the most part, structurally independent from domains in the same sequence. Finally, the structural differences and the lack of interdependence between disordered and structured regions supports that they can be influenced differently by biophysical and structural constraints. For example, an increased purifying selection for protein stability is expected to impact buried residues more than disordered ones. This idea led us to examine whether abundance impacts the relative conservation between these regions. Surprisingly, however, the relative conservation between different regions appeared independent from abundance.

## Results and Discussion

### Disordered Regions Are Equivalent to Super-Accessible Surface Residues in Terms of Their Conservation

Among proteins that need to fold into stable structures to function, amino-acid residues buried in the protein interior contribute the most to stability. Consequently, these residues are under stronger purifying selection than surface amino-acid residues, and are, on average, more conserved in the sequence. Two measures of residue burial have been associated with the heterogeneity of conservation in sequences: (i) solvent accessible surface area or ASA (Lee and Richards, 1971; Shrake and Rupley, 1973; Goldman et al., 1998; Bloom et al., 2006; Lin et al., 2007; Conant and Stadler, 2009; Franzosa and Xia, 2009), which measures the surface or fractional surface of an amino-acid residue that is in contact with bulk water, and (ii) the packing density of an amino-acid residue, which measure the density of its neighbors. Different metrics capture this information, including the contact number and the weighted contact number, with the latter containing longer-range information (Franzosa and Xia, 2009; Yeh et al., 2014). While not strictly equivalent, both accessible surface area and packing density correlate strongly (Echave et al., 2016), and both measures show that the less buried is a residue, the less conserved it is within a protein sequence.

This conservation-structure relationship prompts us to infer structural properties of disordered regions from their pattern of conservation within proteins. We know that disordered regions are devoid of a hydrophobic core and therefore cannot autonomously adopt a stable three-dimensional structure. However, if we consider the spectrum of solvent accessibility and packing density found among folded domains, where would disordered regions position themselves on average? Would they appear much less conserved than even the most solvent-exposed regions? Some disordered regions serve purely as linkers or entropic springs and are expected to show very weak sequence conservation (Dyson and Wright, 2005; Van der Lee et al., 2014). At the same time, disordered regions can also form secondary structure elements and bind to partners (Tompa, 2005; Vacic et al., 2007; Uversky and Dunker, 2010; Wright and Dyson, 2015; Banani et al., 2017; Dignon et al., 2019), thereby burying residues and transiently increasing their packing density. For example, p27Kip1 can wrap around the structure of Cdk2 to regulate its function (Russo et al., 1996; Galea et al., 2008).

**FIGURE 1:**
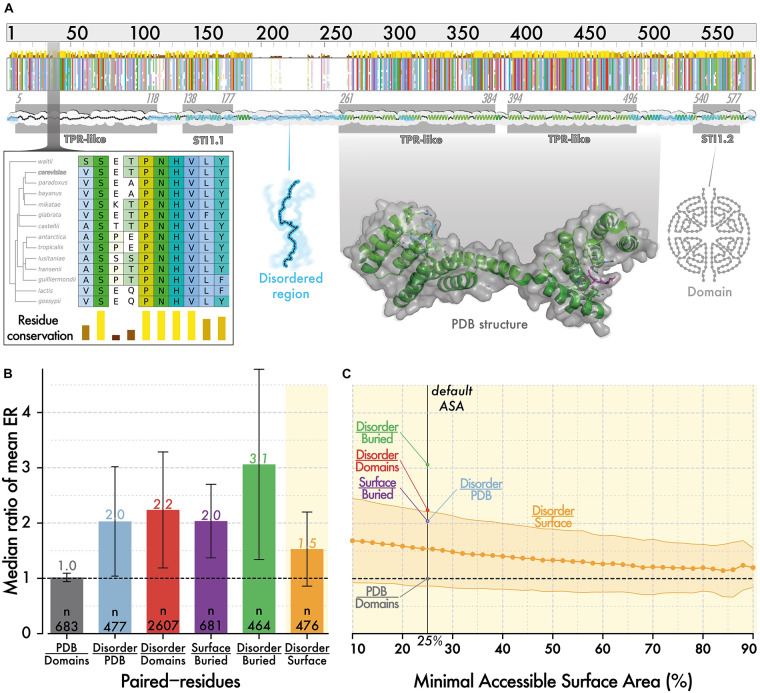
The evolutionary rate of disordered regions is comparable to that of super-exposed regions in folded proteins. **(A)** Evolutionary information and structural features are mapped onto protein sequences from *S. cerevisiae*. The minimap represents the multiple sequence alignment of orthologous sequences to STI1. The amino acids are colored using CLUSTAL’s color scale (Thompson et al., 1994) depending on residue type and conservation. The zoomed-in panel illustrates residue-level conservation, which we calculated with Rate4Site (Pupko et al., 2002). We mapped the positions of PFAM (Bateman et al., 2002) and SUPERFAMILY (Gough and Chothia, 2002) domains (gray box), and of disordered regions predicted by IUPRED (Dosztányi, 2018) (cyan ribbon). We also mapped structural information available from PDB (Rose et al., 2017; Armstrong et al., 2019) and 3DComplex (Levy et al., 2006) on sequences. For this particular sequence, structural information was partially available based on PDB code 3UQ3 (Schmid et al., 2012). **(B)** Within proteins, the evolutionary rate of residues in different regions are averaged, and we compare the ratio of these averages. We show the median of ratios with error bars corresponding to the median absolute deviation. Surface and buried residues are defined based on relative ASA of >25 and ≤25%, respectively (Levy, 2010). **(C)** We calculate the same ratio as in panel **(B)**, between disordered regions and surface regions, using an increasingly stringent relative ASA cut-off to define surface residues. As we increase the cutoff, the median ratio tends toward 1, which highlights that disordered residues evolve only slightly faster than the most exposed residues at protein surfaces.

To position disordered regions on the solvent accessibility spectrum observed in structured regions, we compared the evolutionary rate of residues in both region types. Specifically, we selected 3,350 proteins from *Saccharomyces cerevisiae*, which contain at least 20 residues in both structured regions and disordered regions. We inferred residue-level conservation using Rate4Site (Pupko et al., 2002) on multiple sequence alignments of orthologs from 14 fungal species (see section “Materials and Methods”). Evolutionary and structural information were mapped along the reference sequence from the multiple alignment as illustrated for STI1, a conserved Hsp90 co-chaperone (Figure 1A). We calculated a ratio per protein *i*, corresponding to the mean evolutionary rate of residues in disordered regions (*R*_*i*_^*diso*^) divided by the mean rate of residues in a domain (*R*_*i*_^*domain*^). Overall, considering 2607 proteins with known orthologs, containing both types of regions, the median ratio (*R*_*i*_^*diso*^*^/^R_*i*_*^*domain*^) is equal to 2.2 (Figure 1B). If we now consider domains of known structure (i.e., present in PDB, currently ∼670) instead of those predicted, we find a similar median ratio equal to 2.0. For those proteins, we compared the conservation of disordered regions to that of buried and surface residues separately and found ratios equal to 3.1 and 1.4, respectively. Thus, in an average protein of this dataset, disordered regions evolve 3.1 and 1.4-fold faster than buried and surface residues, respectively (Figure 1B).

This result is based on a definition of surface that includes residues with >25% relative ASA. As higher ASA is associated with lower conservation, we asked whether increasing the cut-off progressively from >25 to >80% would yield a point where surface residues evolve faster than disordered ones (Figure 1C). We did not reach such a point as the ratio remained above 1 for all values. However, the ratio did converge to a value close to 1, highlighting that in an average protein, disordered residues are almost equivalent in their conservation to the most exposed residues at the surface of structured regions.

If we assume that the differential conservation of sites within protein sequences largely reflects different structural constraints, we can infer that disordered regions are, on average, highly solvent-exposed and under weak structural constraints. In sum, our results place disordered regions in the continuum of protein structure, at the end of the solvent-accessibility spectrum. It will be interesting to refine this relationship in the future. For example, by comparing additional properties such as hydrophobicity (Kyte and Doolittle, 1982) or stickiness (Levy, 2010), by considering where disordered segments fall in the sequence (e.g., N/C-ter and inside domains), or by breaking down disorder into different types (Bellay et al., 2011).

### Conservation of Disorder Versus Domains Is Poorly Correlated Among Low Abundance Proteins and the Correlation Increases With Abundance

**FIGURE 2:**
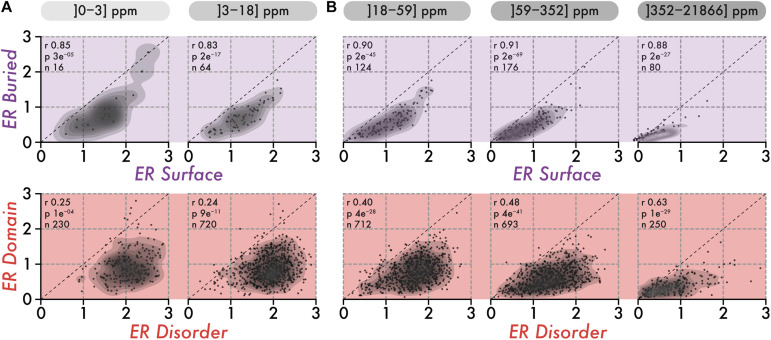
The correlation in the conservation of disorder vs domain regions is poor among low abundance proteins and increases with abundance. **(A)** The top row shows the average evolutionary rate (ER) of surface residues (*x*-axis) vs buried residues (*y*-axis) per protein, for two classes of abundance (0–3 and 3–18 ppm or parts per millions). The lower row shows the average ER of disordered residues (*x*-axis) vs residues in domains (*y*-axis) per protein, for the same two classes of abundance. A protein falling on the diagonal (dashed line) means that residues in the two regions being compared have equal evolutionary rates (i.e., a ratio of 1). The Spearman rank correlation coefficient (r), the associated *p*-value (*p*, two-sided Spearman’s rank correlation test), and the number of proteins (n) within each class of abundance are given above each scatterplot. **(B)** Same as in panel **(A)**, for three classes of abundance (18–59, 59–352, and 352–21,866 ppm or parts per million).

Individual residues within a structure contribute to stability together. As a result, we can expect the evolutionary conservation of residues within a structure to be uniform. To examine this idea, we compared the average evolutionary rate of surface and buried amino-acid residues within structures. Importantly, we know that protein abundance imposes global constraints on the conservation of proteins, which may also result in a uniform evolutionary pressure across the sequence, independently of the structure. Thus, we initially focused on low abundance proteins in which such global constraints are minimized. We observed the conservation of surface and buried regions to correlate strongly (*R* > 0.83, Figure 2A), which is reminiscent of the surface-core association described previously (Tóth-Petróczy and Tawfik, 2011).

We next compared the association in evolutionary conservation between disordered regions and domains found in the same protein. In this case, the correlation was reduced greatly (*R* = 0.25), indicating that the structural connectivity and interdependence between disordered regions and domains are globally weak. These results are consistent with those of the previous section, which depict disordered regions as being highly solvent-accessible and structurally independent from domains. However, proteins expressed at higher levels show increasing correlation, from *R* = 0.40 among medium abundance proteins, to *R* = 0.63 in the class of proteins with the highest abundance (Figure 2B, lower row). This apparent coupling in evolutionary rates is unlikely to have a structural origin. Rather, it probably results from global constraints linked to abundance and exerted on the whole protein sequence. This apparent coupling also implies that different regions in a sequence all experience increasingly strong purifying selection with increasing abundance. This observation led us to quantify whether such negative selection increases equally in all regions, or whether some regions become more constrained than others.

### Evolutionary Constraints Imparted by Protein Abundance Scale Similarly Among Surface, Buried, and Disordered Regions

We saw that surface residues in a protein evolve twice as fast as buried residues on average. This difference, which has long been recognized, is mainly explained by solvent-accessibility/packing density and reflects that protein structures are more likely to be destabilized by mutations at buried positions than by mutations at the surface (Koshi and Goldstein, 1995; Goldman et al., 1998; Guo et al., 2004; Bloom et al., 2006; Sasidharan and Chothia, 2007; Goldstein, 2008; Conant and Stadler, 2009; Franzosa and Xia, 2009; Liberles et al., 2012; Yeh et al., 2014; Echave et al., 2015; Shahmoradi and Wilke, 2016; Spielman and Wilke, 2016; Echave and Wilke, 2017; Liu et al., 2017). Similarly, residues in disordered regions evolve faster than those in domains. Interestingly, this reflects that surface, buried, and disordered residues experience different structural and biophysical constraints. Thus, we propose to examine whether the ratio of their conservation is changing as a function of abundance. For example, observing that buried residues are twice more conserved than surface residues among low abundance proteins, and become four-times more conserved among high abundance proteins would suggest that stability is increasingly constrained with higher abundance.

**FIGURE 3:**
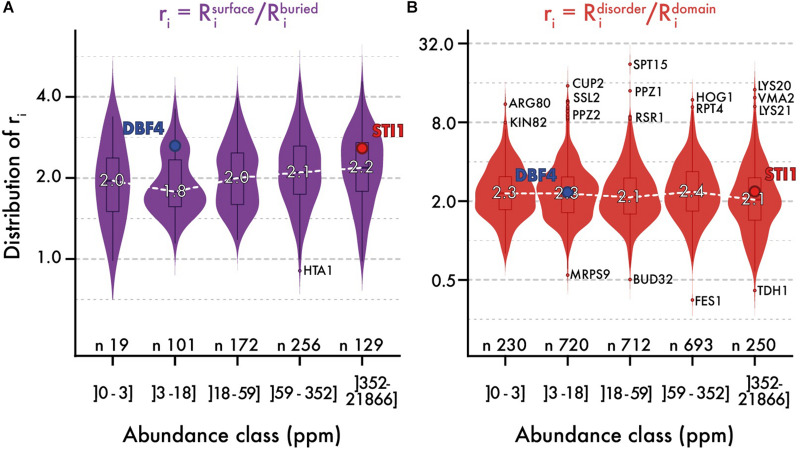
The relative evolutionary rates of different protein regions are steady with abundance. Distribution of evolutionary rates ratio between different regions in the sequence (*y*-axis), across five classes of protein abundance (*x*-axis). A ratio is calculated by dividing the average evolutionary rate of residues found in two regions panel **(A)** surface vs. buried, panel **(B)** disorder vs. domain. The white dashed line highlights the median ratio across bins of abundance. Overlaid box plots show the interquartile range (IQR = 25 to 75% quantiles) with their whiskers extending to 1.58 × IQR. Beyond this interval, the three most extreme outlier values are annotated. The number of proteins contributing to each distribution is given. We also highlight the relative rates for a pair of proteins, one with low and one with high abundance (STI1 and DBF4). These two proteins show comparable structural features, different evolutionary rates (respectively, 0.575 and 1.34 for their full sequence), and similar ratios.

**FIGURE 4:**
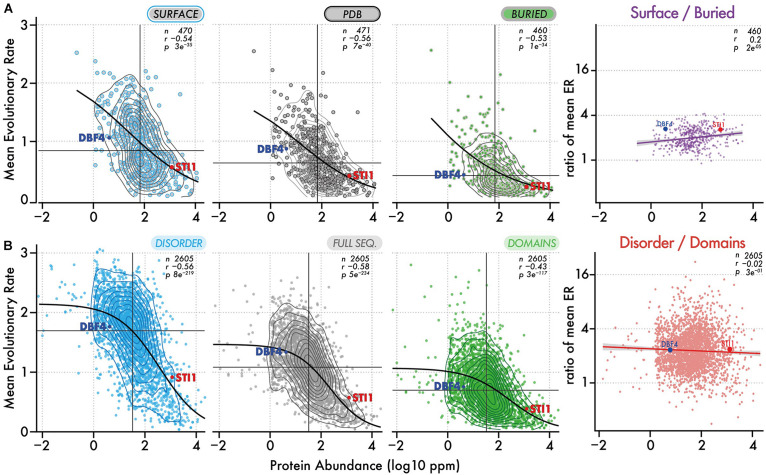
Evolutionary rates of different regions and their ratio as a function of abundance. **(A)** Evolutionary rates (*y*-axis) as a function of protein abundance (*x*-axis) for surface regions, full-length structures, and buried regions. The ratio of evolutionary rate for surface vs buried regions is also shown as a function of abundance. Contour lines show the density of points. The median evolutionary rate and median protein abundance are shown by a vertical and horizontal line, respectively. The Spearman rank correlation coefficient and *p*-value are given with the number of proteins in each dataset. A black line shows the fitted sigmoidal regression for each plot. We highlight two proteins, one with a low and one with a high abundance (DBF4 and STI1). Both have comparable structural features but different evolutionary rates. **(B)** Same representation as in panel **(A)**, now considering disordered versus domain regions.

We analyzed the ratio of conservation (Figures 3A, 4A) of surface and buried residues as a function of abundance. The distribution of these ratios showed comparable median values of about ∼2. In the highest abundance class, this ratio reached ∼2.2 (Figure 3A) creating a significant albeit weak (*R* = 0.2) correlation (Figure 4A). Overall, the ratio is relatively stable, implying that both regions are constrained to a similar extent with increasing abundance. Alternatively, a relatively constant ratio could be favored by the coupling we observed between interior and surface regions (Figure 2, top row). Accordingly, constraints placed on the protein surface could percolate to interior regions and vice versa (Tóth-Petróczy and Tawfik, 2011). To control for this effect, we next compared disordered and domain regions, which show minimal structural coupling. We also observed a stable ratio of ∼2 across the five same abundance classes (Figure 3B), and we observed no dependence of the ratio with abundance even at the highest levels (*R* = −0.02, Figure 4B). Additionally, focusing on disorder and domain regions increased the size of the dataset as we were not limited by the availability of atomic-resolution structures, so this observation applies to the yeast proteome.

By definition, disordered regions and domains should experience distinct structural and biophysical constraints. Thus, the fact that these two regions appear equally constrained with increasing abundance is puzzling and can be interpreted in different ways. One possible explanation is that constraints associated with abundance apply to entire sequences independently of structure. Such constraints could include translational selection (Akashi, 2003), although region-specific codon-bias constraints may exist as well (Tuller et al., 2010; Pechmann and Frydman, 2013), cost of expression (Dekel and Alon, 2005; Wagner, 2005; Cherry, 2010; Gout et al., 2010; Plata et al., 2010), as well as other functional elements and sequence properties that may impact transcription or translation (Stergachis et al., 2013; Zhou et al., 2016). Alternatively or in addition, region-specific structural and biophysical constraints associated with protein concentration could increase in similar proportions with abundance, resulting in a stable ratio. In this case, two primary constraints have been characterized: a first on protein stability (Serohijos et al., 2012, 2013) leading to selection against misfolding (Drummond et al., 2005; Drummond and Wilke, 2008), would dominate among interior residues. A second, on protein solubility (Knowles et al., 2014; Garcia-Seisdedos et al., 2017, 2018; Dubreuil et al., 2019; Foy et al., 2019; Macossay-Castillo et al., 2019; Vecchi et al., 2020), with selection against promiscuous interactions (Deeds et al., 2007; Levy et al., 2009, 2012; Liberles et al., 2011; Yang et al., 2012), would dominate among solvent-exposed residues. However, the fact that constraints on different regions scale proportionally with abundance may appear surprising and will need to be explored in future works.

## Conclusion

We analyzed the evolutionary conservation of sites within proteins, and of proteins within proteomes. We found that disordered regions evolve about three-fold faster than buried regions, and 1.4-fold faster than surface regions. Additionally, disordered regions evolve about as fast as the most solvent-exposed surface regions, highlighting that they extend the continuum of protein structure as a “super-accessible” surface. Unlike regular surface residues, however, disordered regions evolve more independently from domains in the same sequence. This independence allowed us to examine how abundance constrains different regions that are not structurally connected in sequences. Notably, the evolution of disordered regions and domains changed in a similar proportion with abundance: on average, disordered regions evolved twice as fast as domains across the entire range of abundance. Since different regions are subject to different structural and biophysical constraints, we foresee that such comparative analyses of conservation-ratios as a function of abundance will help identify mechanisms underlying the abundance-conservation relationship. It is likely that multiple mechanisms are at play (Mehlhoff et al., 2020) and may be captured by targeted analyses of specific regions and protein subsets.

## Materials and Methods

### Reference Proteome Sequences

The sequences were taken from the reference *S. cerevisiae* proteome maintained by SGD (Cherry et al., 2012). To facilitate data integration, we also mapped those reference sequences against the UniprotKB complete proteome for *S. cerevisiae* (Stutz et al., 2006; UniProt Consortium, 2019).

### Crystallographic Structures

We relied on the 3DComplex database (Levy et al., 2006) to map UNIPROT sequences onto atomic coordinates of protein structures. For each yeast protein, the structures matching the UNIPROT sequence with the largest sequence overlap (minimum 20%) and identity above 90% were retained. Only experimentally determined crystallographic structures with resolutions below 3.0 Ångtrsoms were considered.

### Cellular Abundance

Protein abundances were obtained from Pax-Db (v4.0, May 2015) (Wang et al., 2012, 2015), which provides relative abundances for unicellular and multicellular organisms including tissue-specific data. We use overall abundance inferred from all available data sets (integrated data set).

### Orthologs Alignment and Position-Specific Evolutionary Rate

**FIGURE 5:**
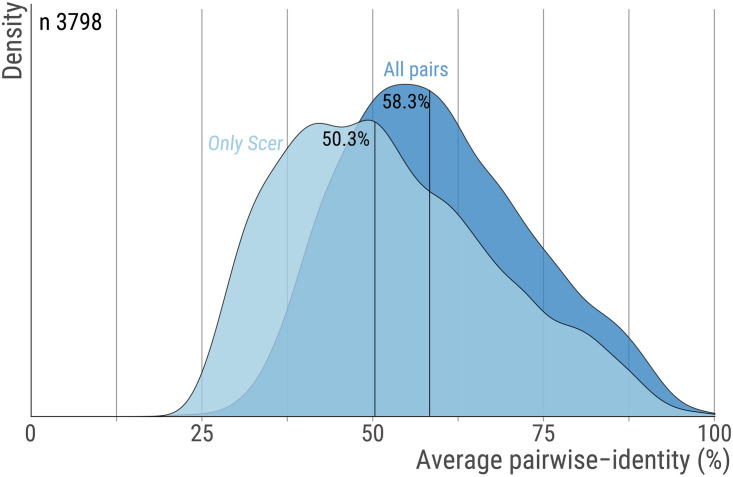
Pairwise sequence identity across orthologs pairs. For each orthogroup we calculate the average percent sequence-identity using all ortholog pairs or only pairs that include the *S. cerevisiae* protein. The distribution for these two measures are shown with dark and light blue, respectively. Vertical lines highlight the median. The number of orthogroups is 3,798.

The orthologs’ alignments were obtained from the original work by Wapinski et al. (2007). Briefly, genes sharing significant sequence similarity were clustered into putative orthogroups and their phylogeny was constructed by a modified neighbor-joining procedure based on pre-computed residues similarities and shared synteny scores. This process was repeated and optimized until each orthogroup consisted of genes that shared a single common ancestor. Here, we used 3798 groups of orthologous proteins along with their multiple sequence alignment encompassing 14 fungal species (*S.cerevisiae, Saccharomyces paradoxus, Saccharomyces mikatae, Saccharomyces bayanus, Naumovozyma castellii (Saccharomyces castellii), Candida glabrata, Kluyveromyces lactis, Debaryomyces hansenii, Yarrowia lipolytica, Eremothecium gossypii (Ashbya gossypii), Lachancea waltii (Kluyveromyces waltii), Candida albicans, Aspergillus nidulans, Fusarium graminearum, Magnaporthe grisea, Neurospora crassa, Cryptococcus neoformans, Schizosaccharomyces pombe*) were used. Only 6 orthogroups had one sequence missing and these were replaced by indels. The median pairwise sequence identity within these 3,798 orthogroups is 58.3% (Figure 5).

All alignments were computed using MUSCLE (Edgar, 2004) and then concatenated to estimate residue-level evolutionary rate using the software Rate4Site (Pupko et al., 2002). Additional details on how evolutionary rates were estimated are available in Landry et al. (2009).

### Intrinsic Disorder Predictions

We predicted disordered regions in the yeast proteome by combining short and long disorder segments predicted by IUPred (Mészáros et al., 2009; Dosztányi, 2018). We considered the 20% amino-acid residues with the highest disorder probabilities among all proteins. In all analyses, we required a minimum number of 20 residues in a particular region to calculate an average evolutionary rate. When fewer residues were available, the average rate of the region was considered undefined.

### Domains Assignment

To assign domains, we aligned profiles from Pfam-A (v27.0, May 2013) (Bateman et al., 2002; Finn et al., 2014) and SUPERFAMILY (v1.75, March 2013) (Gough, 2002; Oates et al., 2015) to reference proteome sequences, filtering the hits with an *E*-value score above 10^–3^. Finally, domain residues are those that were identified as part of a hit from either Pfam, SUPERFAMILY, or both.

## Data Availability Statement

The original contributions presented in the study are included in the article/supplementary material, further inquiries can be directed to the corresponding author/s. Data used in this work are available on Figshare in a tabulated format: https://doi.org/10.6084/m9.figshare.13738657.

## Author Contributions

BD and EL designed the analyses and experiments, analyzed the data, and wrote the manuscript. BD carried out the analyses. Both authors contributed to the article and approved the submitted version.

## Conflict of Interest

The authors declare that the research was conducted in the absence of any commercial or financial relationships that could be construed as a potential conflict of interest.
